# Effects of compound probiotics on intestinal and liver injury in Lohmann Pink chickens challenged by lipopolysaccharide

**DOI:** 10.1016/j.psj.2026.107306

**Published:** 2026-06-17

**Authors:** Yongliang Zhang, Xixi Dai, Bingran Yu, Jia Yu, Linlin Wang, Ruoxi Zhan, Liangqing Chen, Xing Liu, Kaiqing Rao

**Affiliations:** a College of Animal and Veterinary Sciences, Southwest Minzu University, Chengdu 610041, China; b Key Laboratory of Animal Medicine of Sichuan Education Department, Southwest Minzu University, Chengdu 610041, China; c Chongqing Three Gorges Vocational College, Chongqing 404155, China

**Keywords:** Compound probiotics, Lohmann Pink chickens, Lipopolysaccharide, Intestinal barrier, Liver injury

## Abstract

This study aimed to explore the effects of dietary compound probiotics (*Bacillus subtilis, Bacillus coagulans, Saccharomyces cerevisiae*) supplementation on intestinal and liver injury induced by lipopolysaccharide (LPS) in Lohmann Pink chickens. A total of twenty-four eight-week-old healthy commercial Lohmann Pink chickens were randomly allocated to three groups (n = 8 per group, half males and females in each group): Control group (basal diet + saline injection), LPS group (basal diet + LPS injection), and LPS+Pro group (basal diet supplemented with 0.2 g/kg compound probiotics + LPS injection). On days 15, 17, and 19 of the experiment, chickens in the LPS and LPS+Pro groups were challenged with LPS (0.3 mg/kg) via intraperitoneal injection, while the Control group received an equivalent volume of sterile saline. Sampling was performed on day 22 of the experiment. Results showed that LPS challenge markedly decreased average daily gain (ADG) (*P* < 0.05), increased rectal temperature and spleen index (*P* < 0.05), and caused mucosal damage of the duodenum and jejunum. LPS significantly elevated serum diamine oxidase (DAO) and alanine aminotransferase (ALT) activities, as well as D-lactic acid (D-LA) and LPS concentrations (*P* < 0.05), while reducing the mRNA expression of intestinal tight junction genes *Claudin-1* and *ZO-1* (*P* < 0.05). Meanwhile, LPS significantly increased malondialdehyde (MDA) content (*P* < 0.05) and decreased superoxide dismutase (SOD), glutathione peroxidase (GSH-Px), and catalase (CAT) activities in serum and liver (*P* < 0.05), accompanied by down-regulated *Nrf2* (*P* < 0.05) and up-regulated *Keap-1, TLR4, NF-κB, TNF-α,* and *IL-6* expression (*P* < 0.05). In contrast, compared with the LPS group, the LPS+Pro group significantly increased ADG, villous height-to-crypt depth ratio (V/C ratio), and intestinal tight junction gene expression (*P* < 0.05), and reduced serum DAO, D-LA, ALT, and LPS levels (*P* < 0.05). The LPS+Pro group also mitigated oxidative stress by normalizing MDA content and CAT activity in the serum and liver, and restored the expression of *TLR4, NF-κB, TNF-α,* and *IL-6* to the Control levels. In conclusion, dietary compound probiotics mitigated LPS-induced intestinal and liver injury in Lohmann Pink chickens by enhancing intestinal barrier integrity and hepatic antioxidant capacity, showing a trend to maintain the homeostasis of the Nrf2/Keap-1 mediated antioxidant pathway and suppressing TLR4/NF-κB mediated inflammatory responses in the liver at the transcriptional level, providing a promising strategy for antibiotic alternatives in healthy poultry breeding.

## Introduction

The abuse of antibiotics in poultry farming has led to multifaceted hazards. Firstly, antibiotic residues in meat and egg products may trigger allergic reactions, toxic effects, and intestinal flora dysbiosis in consumers, and simultaneously facilitate the emergence and transmission of antibiotic-resistant bacteria, posing a severe threat to public health (Izah et al., 2025; Mund et al., 2017). Antibiotics incompletely metabolized in poultry are excreted via feces into the environment, resulting in the spread of antibiotic resistance genes and antibiotic-resistant bacteria in soil and water bodies, which exacerbates environmental pollution and the problem of antimicrobial resistance (Muhammad et al., 2020). Thus, the exploration of alternative antibacterial and anti-inflammatory agents is an urgent necessity.

Probiotics possess multiple functions: they effectively inhibit the growth and proliferation of pathogenic bacteria in the gastrointestinal tract, thereby alleviating symptoms associated with gastrointestinal diseases, enhancing host immune function, and further improving the overall health status of animals (Ohashi and Ushida, 2009), exhibiting excellent potential as antibiotic alternatives.

*Bacillus coagulans* can increase the body weight, average daily gain (ADG), egg weight, and egg production rate of broilers and laying hens, improve feed conversion efficiency, enhance antioxidant capacity and immune function, optimize intestinal structure, and effectively alleviate intestinal damage and inflammation induced by pathogenic bacteria (Wang et al., 2023; Zhang et al., 2021).

Supplementation with *Bacillus subtilis* significantly improves the growth performance, body weight, and ADG of broilers, enhances feed conversion efficiency, and boosts immunity and antioxidant capacity. Additionally, it optimizes the intestinal microbial structure by increasing the abundance of beneficial bacteria such as *Lactobacillus* and *Bifidobacterium*, reducing pathogenic bacteria, improving intestinal morphology and barrier function, and promoting intestinal health (Qiu et al., 2021; Wang et al., 2022).

Yeast and its derivatives, as poultry feed additives, exert multiple significant effects. Yeast can improve the growth performance, feed conversion efficiency, egg weight, and egg production rate of broilers and laying hens, while enhancing immune function, antioxidant capacity, intestinal health, and microbial diversity (Bilal et al., 2022). Active components in yeast cell walls, such as mannan and β-glucan, regulate intestinal flora, promote the growth of beneficial bacteria, inhibit pathogenic bacteria, enhance intestinal barrier function, and reduce inflammatory responses (Bilal et al., 2022).

However, single probiotic strains often have limited functions and unstable effects. Compound probiotics, through the synergistic effects of multiple strains, demonstrate more prominent advantages in improving intestinal health, enhancing immunity, and alleviating stress (Kwoji et al., 2021). In our previous preliminary experiments, the compound probiotic combination of *Bacillus coagulans, Bacillus subtilis*, and *Saccharomyces cerevisiae* significantly improved the growth performance and antioxidant capacity of Lohmann Pink chickens during rearing.

Lipopolysaccharide (LPS), a major component of the cell wall of Gram-negative bacteria, is a classic model for inducing immune stress. Studies have shown that LPS can activate the Toll-like receptor 4 (TLR4) signaling pathway, promote the release of pro-inflammatory cytokines such as tumor necrosis factor-α (TNF-α) and interleukin-1β (IL-1β), trigger systemic inflammatory responses and oxidative stress, and cause significant damage, particularly to the intestine and liver, thereby inhibiting animal growth and reducing production performance (Lu et al., 2008; Luo et al., 2025; Zamyatina and Heine, 2020).

Lohmann Pink chickens are an important breed in the laying hen industry, and their health and production performance directly affect breeding benefits. During the brooding and pullet stages, the immune system of chickens is not fully mature, making them susceptible to invasion by endotoxins such as LPS in the environment. This leads to persistent inflammation and oxidative damage, resulting in impaired growth, decreased egg production performance, and substantial economic losses (Han et al., 2020; Jiang et al., 2019).

Therefore, this study established an LPS-induced immune stress model to systematically investigate the effects of compound probiotics (*Bacillus coagulans, Bacillus subtilis*, and *Saccharomyces cerevisiae*) on intestinal and liver damage in eight-week-old Lohmann Pink chickens, and to explore the underlying mechanisms. The findings may provide a promising strategy for antibiotic alternatives in healthy poultry breeding.

## Materials and methods

### Ethics statement

The experiment was conducted at the experimental farm of the College of Animal and Veterinary Sciences, Southwest Minzu University. All experimental procedures and animal care protocols were approved by the Institutional Animal Ethics Committee of Southwest Minzu University (approval number: SMU-202601070).

### Compound probiotics

The compound probiotics used in this study were a commercial feed additive (Luodong A-90, Fujian Luodong Biotechnology Co., Ltd.), powdered with glucose as the carrier (the daily glucose intake from the carrier in the study was negligible). According to the manufacturer, the active ingredients were *Bacillus subtilis subsp. natto* BNLD (CCTCC M2016442) ≥ 1.0 × 10⁹ CFU/g, *Bacillus coagulans* LDC0429 (CCTCC M2016440) ≥ 1.0 × 10⁸ CFU/g, and *Saccharomyces cerevisiae* (strain Boulardii) ≥ 2.0 × 10⁷ CFU/g. Species identity and viable counts of all three strains were independently verified by a third‑party testing institution (Centre Testing International (Qingdao) Co., Ltd., Report No. A2250113369101001Cb) with the measured values of 2.1 × 10^9^ CFU/g, 2.4 × 10^9^ CFU/g, and 1.8 × 10^9^ CFU/g, respectively. The additive was used within its shelf life and stored according to the manufacturer’s instructions (ventilated, dry, and dark storage); the product was directly mixed into the feed before each feeding, minimizing viability loss.

### Experimental design and husbandry

A total of twenty-four non-vaccinated eight-week-old healthy commercial Lohmann Pink chickens with similar initial body weight (330.47 ± 33.00 g, uniformity ≥ 80%) were randomly divided into three groups (n = 8 per group, half males and females in each group): Control group, LPS group, and LPS+Pro group. The experimental birds underwent prior restricted feeding and consequently exhibited reduced growth rates. One week before the formal experiment, they were acclimated to a basal diet (Table S1) for seven days, during which flock uniformity exceeded 80%, and all individual body weights fell within ± 10% of the mean, meeting the requirements for experimental use. The experimental period lasted 22 d. During the trial, the Control and LPS groups received the basal diet, whereas the LPS+Pro group received the basal diet supplemented with 0.2 g/kg compound probiotics. The supplemental dose of compound probiotics was determined based on our preliminary experiments, which exerted significant growth-promoting and antioxidant effects in Lohmann Pink chickens. On days 15, 17, and 19, chickens in the LPS and LPS+Pro groups were intraperitoneally injected with 1 mL LPS (*Escherichia coli* O55:B5, Beijing Labgic Technology Co., Ltd.) solution at a dose of 0.3 mg/kg body weight to induce acute immune stress, while the Control group received an equal volume of sterile saline.

The experiment was conducted in the animal experimental facility of Southwest Minzu University, Sichuan Province. The ambient temperature was maintained at 24∼25 °C, relative humidity at 50∼60%, with a 14 h light : 10 h dark photoperiod. Each group was raised in a cage (1 m × 0.5 m × 0.5 m), and all chickens were individually numbered for the recording of average daily gain and individual biochemical indices. Feed and water were provided ad libitum throughout the trial. The basal diet (grower feed) was formulated according to the Lohmann Pink Laying Hen Feeding Manual (07.22_V07-23), and its composition and nutrient levels are presented in Table S1.

### Sample collection

On the morning of day 22 of the experiment, all chickens were weighed and euthanized for sample collection. The liver, bursa, spleen, and heart were excised and weighed (Precisa PT620M balance, Switzerland), and relative organ weights were calculated as (organ weight / body weight) × 100, expressed as percentages. Blood samples were collected from the jugular vein, incubated in a 37 °C thermostat for 1 h, and centrifuged at 3000 r/min for 15 min to separate serum. The serum was stored at −20 °C until analysis. Meanwhile, liver, duodenum, and jejunum samples were collected into cryovials, immediately snap-frozen in liquid nitrogen, and then stored at −80 °C for subsequent assays. Tissue samples from identical anatomical sites, specifically 3 cm segments of the duodenum and jejunum, as well as the right liver lobe, were rinsed gently with cold saline and fixed in 4% paraformaldehyde. Samples for hematoxylin-eosin (HE) staining were randomly selected within each group, while samples for biochemical and gene expression indicators were chosen with equal numbers of males and females in each group to minimize potential sex‑related effects.

### Average daily gain and rectal temperature

All chickens were individually numbered. Body weight was recorded at weeks 0, 1, 2, and 3 during the trial, and ADG of each group was calculated accordingly. Three chickens were randomly selected from each group for rectal temperature measurement, which was performed daily for one week before and one week after the first challenge. Rectal temperature was measured using a mercury thermometer inserted 2 cm into the rectum until a stable reading was obtained.

### Serum and liver antioxidant indicators

After defrosting, 1 g of chicken liver tissue was homogenized at 8,000 × g for 10 s in 9 mL of 0.9% saline buffer under an ice bath. The homogenate was then centrifuged at 3,000 × g at 4 °C for 10 min, and the supernatant was collected for subsequent analysis.

The supernatant and collected serum were used to determine the levels of superoxide dismutase (SOD, A001-3), glutathione peroxidase (GSH-Px, A005-1), catalase (CAT, A007-1-1), and malondialdehyde (MDA, A003-1) according to the manufacturer’s instructions (Nanjing Jiancheng Bioengineering Institute, China). Protein concentration of liver supernatants was quantified using the Coomassie Brilliant Blue method, with absorbance recorded at 595 nm. SOD activity was determined by the Water-Soluble Tetrazolium Salt-1 method with absorbance detected at 450 nm. GSH-Px activity was assayed in a GSH-H₂O₂ system, where remaining GSH reacted with DTNB and was read at 412 nm. CAT activity was assayed based on H₂O₂ decomposition; the remaining H₂O₂ reacted with molybdate to form a colored complex detected at 405 nm. Lipid peroxidation was quantified as MDA content using the thiobarbituric acid method: after boiling and blank correction, absorbance was measured at 532 nm. Absorbance values were measured using a microplate reader (SpectraMax iD3, Molecular Devices).

### Serum biochemical indicators

The collected serum was used to determine the levels of alanine aminotransferase (ALT, C009-1-1), aspartate aminotransferase (AST, C010-1-1), diamine oxidase (DAO, A088-3-1), and D-lactic acid (D-LA, A019-3-1) following the manufacturer’s instructions (Nanjing Jiancheng Bioengineering Institute, Nanjing, China). ALT and AST activity was measured by a colorimetric method in which the produced pyruvate reacted with 2,4‑dinitrophenylhydrazine to form a reddish‑brown product, with absorbance detected at 505 nm. D‑LA content was determined by an enzymatic method where D‑lactate was converted by D‑lactate dehydrogenase to generate NADH, which then reacted with a specific chromogen to form a colored product measured at 450 nm. DAO activity was assayed using a kinetic method coupled with a glutamate dehydrogenase reaction that consumed NADH, and the activity was calculated from the decrease in absorbance per minute (ΔA/min) at 340 nm. The liver supernatant and serum LPS levels were measured using a commercial ELISA kit (MM-37108O2, Jiangsu Meimian Industrial Co., Ltd., Yancheng, China) in strict accordance with the manufacturer’s protocols (two-step double-antibody sandwich method, absorbance was measured at 450 nm). Absorbance values were measured using a microplate reader (SpectraMax iD3, Molecular Devices).

### Duodenal, jejunal, and hepatic morphology

Duodenum, jejunum, and liver tissues fixed in 4% paraformaldehyde were subjected to paraffin embedding, section preparation, and HE staining by Wuhan Boerfu Co., Ltd., China. Images were captured using an optical microscope (ZEISS, Germany). For intestinal tissues, intact visual fields from the same anatomical position were selected, and ImageJ software was used to quantitatively analyze villus height, crypt depth, and the villus height to crypt depth ratio (V/C ratio). For liver tissues, three non-overlapping visual fields were randomly selected per sample, and representative images were collected for subsequent analysis.

### Quantitative real-time PCR analysis

Total RNA was extracted from the collected duodenum, jejunum, and liver tissues using TRIzol reagent (RNAiso Plus, Takara China, Dalian, China). RNA concentration and purity were evaluated by measuring the A260/A280 absorbance ratio using a NanoDrop ONEC spectrophotometer (Hongzhong Medical Equipment Co., Ltd.). RNA integrity was verified by 1% agarose gel electrophoresis. First-strand cDNA was then synthesized from 1 µg of total RNA using a reverse transcription kit containing a genomic DNA (gDNA) removal step (RR092A, Takara, Dalian, China) in accordance with the manufacturer’s instructions. The qPCR assay was performed using TB Green Taq Fast qPCR Mix (CN830A, Takara, Dalian, China) on a QuantStudio 3 real-time PCR system (Thermo Fisher Scientific, China). Amplification specificity was confirmed by melting curve analysis, with a single distinct peak detected for each amplicon. Using *β-actin* as the internal reference gene, the relative mRNA expression levels of target genes were determined, including hepatic antioxidant-related genes: Superoxide dismutase (*SOD*), Glutathione peroxidase 1 (*GPX1*), Nuclear factor erythroid 2-related factor 2 (*Nrf2*), Kelch-like ECH-associated protein 1 (*Keap-1*); hepatic immune-related genes: Toll-like receptor 4 (*TLR4*), Nuclear factor kappa-B (*NF-κB*), Interleukin-6 (*IL-6*), Tumor necrosis factor-α (*TNF-α*); intestinal tight junction genes: *Claudin-1*, Zonula occludens-1 (*ZO-1*), *Occludin*. Data were analyzed using the 2^−ΔΔ^*^Ct^* method to determine the relative mRNA abundance of each target gene. Primer sequences are listed in Table 1.

**Table 1:** Primers used for real-time reverse-transcription PCR.

RNA target	GenBank accession no.	Primer sequence
*β-actin*	NM_205518.2	F:TGCGTGACATCAAGGAGAG
		R:TGCCAGGGTACATTGTGGTA
*Nrf2*	XM_046921114.1	F:GGGACGGTGACACAGGAACAAC
		R: GCTCTCCACAGCGGGAAATCAG
*SOD*	NM_205064.2	F: TCTTACCGGACCACACTGCATC
		R: ACGAGGTCCAGCATTTCCAGTTA
*GPX1*	NM_001277853.3	F: GACCAACCCGCAGTACATCA
		R: GAGGTGCGGGCTTTCCTTTA
*Keap-1*	XM_030475226	F: GCATCACAGCAGCGTGGAGAG
		R: GGCGTACAGCAGTCGGTTCAG
*TLR4*	NM_001030693.1	F: TTCGGTTGGTGGACCTGAATCTTG
		R: ACAGCTTCTCAGCAGGCAATTCC
*NF-κB*	NM_001396038.1	F: CCACAACACAATGCGCTCTG
		R: AACTCAGCGGCGTCGATG
*TNF-a*	XM_046927261.1	F: GCCTATGCCAACAAGTACACCT R: GCCAAGTCAACGCTCCTG
*IL-6*	NM_204628.1	F: AAATCCCTCCTCGCCAATCT R: CCCTCACGGTCTTCTCCATAAA
*ZO-1*	XM_015278975	F: GCCAACTGATGCTGAACCAA R: GGGAGAGACAGGACAGGACT
*Claudin-1*	NM_001013611	F: CTGATTGCTTCCAACCAG R: CAGGTCAAACAGAGGTACAAG
*Occludin*	NM_205128.1	F: GATGGACAGCATCAACGACC
		R: CTTGCTTTGGTAGTCTGGGC

### Statistical analysis

All figures and tables were generated using GraphPad Prism 9 software. Data are presented as means ± standard error of the mean (SEM). Statistical analysis was performed using one-way ANOVA with Duncan’s multiple range test in SPSS 27.0. *P* < 0.05 was considered statistically significant.

## Results

### Average daily gain and rectal temperature

**Fig. 1:**
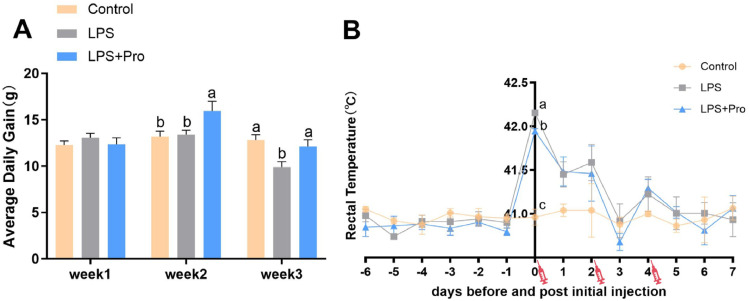
ADG and rectal temperature of Lohmann Pink chickens in the animal experiment. (A) ADG during the animal experiment, n = 8. (B) Rectal temperatures of Lohmann Pink chickens before and after the initial LPS challenge; 0 on x axis indicates 6 h after the first injection; other numbers represent days relative to the first injection, with 24 h intervals, n = 3. Data are presented as mean ± SEM. Bars with different letters (a-c) indicate significant differences (*P* < 0.05). Control: fed a basal diet and injected with saline on days 15, 17, and 19. LPS: fed a basal diet and injected with LPS on days 15, 17, and 19. LPS+Pro: fed a basal diet supplemented with compound probiotics and injected with LPS on days 15, 17, and 19.

In the second week, ADG was significantly higher in the LPS+Pro group (15.96 ± 1.03 g) than in the Control (13.20 ± 0.58 g) and LPS groups (13.39 ± 0.49 g) (*P* *<* 0.05). In the third week, ADG was markedly lower in the LPS group (9.91 ± 0.58 g) than in the Control group (12.81 ± 0.57 g) (*P* *<* 0.05), whereas it was significantly elevated in the LPS+Pro group (12.11 ± 0.73 g) compared with the LPS group (*P* *<* 0.05) and restored to the level of the Control group (*P* *>* 0.05) (Fig. 1A).

Rectal temperature did not differ significantly among groups from day 6 to day 1 before the first LPS challenge. At 6 h after the first LPS injection (day 0), rectal temperature was significantly increased in both the LPS (42.15 ± 0.03 °C) and LPS+Pro groups (41.94 ± 0.03 °C) relative to the Control group (40.96 ± 0.09 °C) (*P* *<* 0.05), while that in the LPS+Pro group was lower than in the LPS group (*P* *<* 0.05). From day 1 to day 7 after the first injection, rectal temperature in all groups stabilized and returned to pre-challenge levels, with no significant differences among groups (*P* *>* 0.05) (Fig. 1B).

### Organ indices

**Fig. 2:**
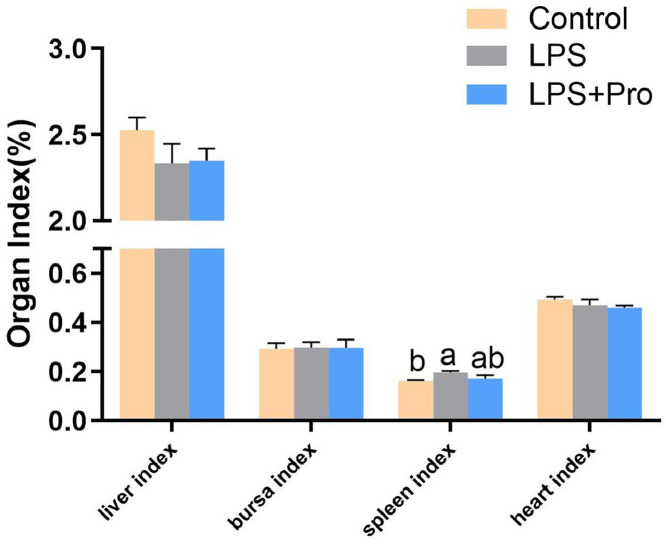
Organ indices of Lohmann Pink chickens on day 22 of the experiment. Data are presented as mean ± SEM, n = 8. Bars with different letters (a-c) indicate significant differences (*P* < 0.05). Control: fed a basal diet and injected with saline on days 15, 17, and 19. LPS: fed a basal diet and injected with LPS on days 15, 17, and 19. LPS+Pro: fed a basal diet supplemented with compound probiotics and injected with LPS on days 15, 17, and 19.

There were no significant differences in heart, liver, and bursa indices among groups (*P* *>* 0.05). The spleen index was significantly increased in the LPS group (0.197 ± 0.006)% compared with the Control group (0.163 ± 0.003)% (*P* *<* 0.05), while no significant differences were observed in the LPS+Pro group (0.172 ± 0.013)% relative to the Control and LPS groups (*P* *>* 0.05) (Fig. 2).

### Duodenal and jejunal morphology

**Fig. 3:**
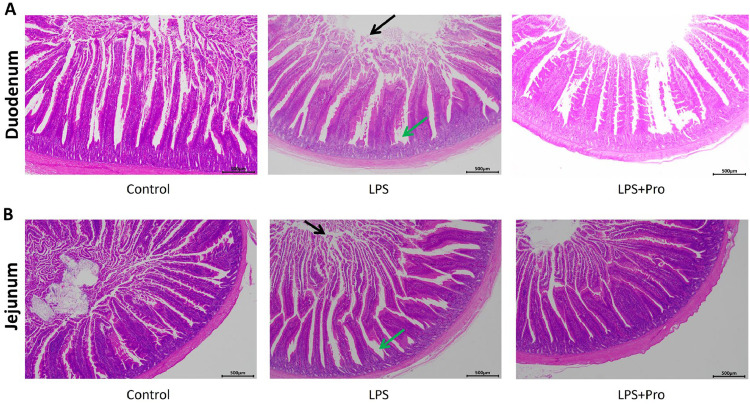
Duodenum and jejunum morphology of Lohmann Pink chickens on day 22 of the experiment. (A) Representative H&E staining images of duodenum sections. (B) Representative H&E staining images of jejunum sections. 40× magnification, Scale bar, 500 μm. The black arrow indicates shedding of epithelial cells, and the green arrow indicates widened intervillous spacing. Control: fed a basal diet and injected with saline on days 15, 17, and 19. LPS: fed a basal diet and injected with LPS on days 15, 17, and 19. LPS+Pro: fed a basal diet supplemented with compound probiotics and injected with LPS on days 15, 17, and 19.

As shown in Fig. 3A, in the Control group, the duodenal mucosa exhibited an intact structure, with neatly arranged and elongated villi, with no obvious pathological changes observed. In the LPS group, the duodenal mucosa displayed significant damage, characterized by shortened and fractured villi, epithelial cell shedding in some regions, and exposed lamina propria, indicating that LPS induced severe disruption of the intestinal mucosal barrier. In contrast, in the LPS+Pro group, duodenal mucosal damage was markedly alleviated: villus morphology was largely restored, the arrangement became relatively orderly, and epithelial cell integrity was enhanced, suggesting that probiotics effectively mitigated LPS-induced duodenal mucosal injury.

As shown in Fig. 3B, the jejunal mucosa of the Control group featured tall, dense, and regularly arranged villi, intact epithelial cells, and normal mucosal architecture. In the LPS group, jejunal villi showed obvious fracture, with epithelial cell shedding in some areas, and compromised integrity of the mucosal barrier. In the LPS+Pro group, jejunal mucosal morphology was significantly improved: the arrangement became more orderly, and epithelial cell shedding was reduced, demonstrating that probiotics effectively protected the jejunal mucosa from LPS-induced damage.

**Fig. 4:**
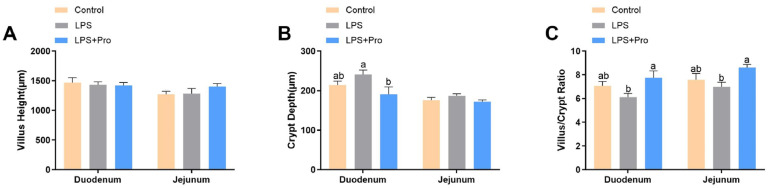
Duodenum and jejunum morphology values of Lohmann Pink chickens on day 22 of the experiment. (A) Villus height. (B) Crypt depth. (C) Villous height to crypt depth ratio. Data are presented as mean ± SEM, n = 5. Bars with different letters (a-c) indicate significant differences (*P* < 0.05). Control: fed a basal diet and injected with saline on days 15, 17, and 19. LPS: fed a basal diet and injected with LPS on days 15, 17, and 19. LPS+Pro: fed a basal diet supplemented with compound probiotics and injected with LPS on days 15, 17, and 19.

As shown in Fig. 4, no significant difference in villus height was observed among all groups in either the duodenum or jejunum (*P* *>* 0.05) (Fig. 4A). In the duodenum, crypt depth was significantly higher in the LPS group than in the LPS+Pro group (*P* *<* 0.05) (Fig. 4B). In both the duodenum and jejunum, villous height to crypt depth ratio (V/C ratio) was significantly higher in the LPS+Pro group than in the LPS group (*P* *<* 0.05) (Fig. 4C).

### Intestinal barrier permeability

**Fig. 5:**
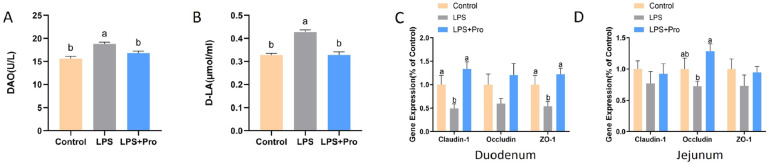
Intestinal permeability-related parameters of Lohmann Pink chickens on day 22 of the experiment. (A) Serum DAO activity. (B) Serum d-lactic acid concentration. (C) Relative mRNA expression of intestinal barrier-related genes in duodenum. (D) Relative mRNA expression of intestinal barrier-related genes in jejunum. Data are presented as mean ± SEM, n = 6. Bars with different letters (a-c) indicate significant differences (*P* < 0.05). Control: fed a basal diet and injected with saline on days 15, 17, and 19. LPS: fed a basal diet and injected with LPS on days 15, 17, and 19. LPS+Pro: fed a basal diet supplemented with compound probiotics and injected with LPS on days 15, 17, and 19.

As shown in Fig. 5, compared with the Control and LPS+Pro groups, the LPS group showed significantly higher serum DAO activity and D-LA concentration (*P* *<* 0.05), while the LPS+Pro group returned to levels comparable to the Control group (*P* *>* 0.05) (Fig. 5A, B). Meanwhile, the mRNA expression levels of *Claudin-1* and *ZO-1* in the duodenum were significantly lower in the LPS group than in the Control group (*P* *<* 0.05) and LPS+Pro group (*P* *<* 0.05) (Fig. 5C). In the jejunum, the mRNA expression level of *Occludin* was significantly increased in the LPS+Pro group compared with the LPS group (*P* *<* 0.05) (Fig. 5D).

### Liver morphology and liver injury-related biochemical indicators

**Fig. 6:**
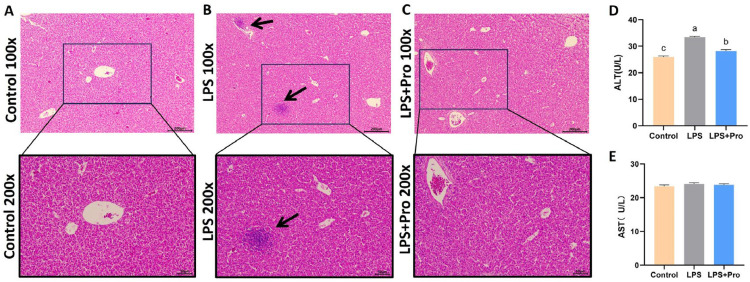
Liver morphology and biochemical indicators of liver injury in Lohmann Pink chickens on day 22 of the experiment. (A) Liver morphology in the Control group. (B) Hepatic morphology in the LPS group. (C) Liver morphology in the LPS+Pro group. HE staining, 100× magnification, scale bar: 200 μm; 200× magnification, scale bar: 50 μm; n = 3. (D) Serum ALT activity. (E) Serum AST activity. Data are presented as mean ± SEM, n = 6. Bars with different letters (a-c) indicate significant differences (*P* < 0.05). The black arrow indicates inflammatory cell infiltration. Control: fed a basal diet and injected with saline on days 15, 17, and 19. LPS: fed a basal diet and injected with LPS on days 15, 17, and 19. LPS+Pro: fed a basal diet supplemented with compound probiotics and injected with LPS on days 15, 17, and 19.

In the Control group, the hepatic tissue structure was intact with clear hepatic lobule boundaries. Hepatocyte cords were radially arranged around the central vein, with normal cell morphology and abundant cytoplasm, and no obvious inflammatory cell infiltration or tissue damage was observed (Fig. 6A). In the LPS group, the most prominent feature was the presence of multiple focal inflammatory cell infiltration (Fig. 6B). In the LPS+Pro group, compound probiotics significantly alleviated LPS-induced liver injury compared with the LPS group. The inflammatory infiltration foci were significantly reduced or disappeared, indicating that probiotics exerted a distinct protective effect against LPS-induced liver damage (Fig. 6C).

As shown in Fig. 6D, serum ALT activity was significantly increased in the LPS and LPS+Pro groups compared with the Control group (*P* < 0.05), and was markedly lower in the LPS+Pro group than in the LPS group (*P* < 0.05). Meanwhile, no significant differences in serum AST activity were observed among all groups (*P* *>* 0.05) (Fig. 6E).

### Antioxidant status

**Fig. 7:**
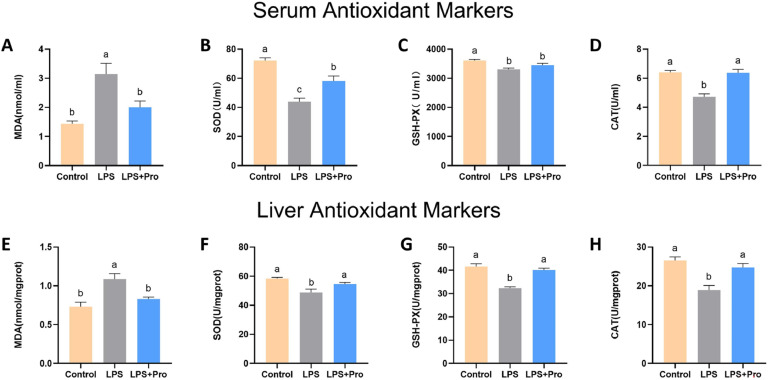
Serum and liver antioxidant status of Lohmann Pink chickens on day 22 of the experiment. (A-D) Serum antioxidant makers. (E-H) Liver antioxidant makers. Data are presented as mean ± SEM, n = 6. Bars with different letters (a-c) indicate significant differences (*P* < 0.05). Control: fed a basal diet and injected with saline on days 15, 17, and 19. LPS: fed a basal diet and injected with LPS on days 15, 17, and 19. LPS+Pro: fed a basal diet supplemented with compound probiotics and injected with LPS on days 15, 17, and 19.

As shown in Fig. 7, compared with the Control group, the serum and liver MDA concentrations in the LPS group were significantly elevated (*P* < 0.05), and the activities of SOD, GSH-Px, and CAT were markedly decreased (*P* < 0.05). Compared with the Control group, the serum MDA concentration and CAT activity in the LPS+Pro group showed no significant differences (*P* *>* 0.05), while the activities of SOD and GSH-Px were significantly reduced (*P* < 0.05). In the liver, however, there were no significant differences in MDA concentration or the activities of SOD, GSH-Px, and CAT between the LPS+Pro group and the Control group (*P* *>* 0.05).

### Hepatic gene expression associated with antioxidant and inflammatory pathways

**Fig. 8:**
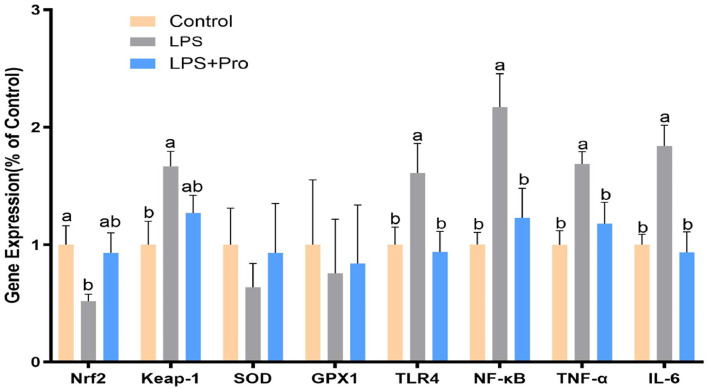
Hepatic relative expression of antioxidant and inflammation related genes in Lohmann Pink chickens on day 22 of the experiment. Data are presented as mean ± SEM, n = 6. Bars with different letters (a-c) indicate significant differences (*P* < 0.05). Control: fed a basal diet and injected with saline on days 15, 17, and 19. LPS: fed a basal diet and injected with LPS on days 15, 17, and 19. LPS+Pro: fed a basal diet supplemented with compound probiotics and injected with LPS on days 15, 17, and 19.

As shown in Fig. 8, in the antioxidant pathway, compared with the Control group, the mRNA expression of *Nrf2* was significantly down-regulated (*P* < 0.05), while that of *Keap-1* was significantly up-regulated (*P* < 0.05) in the LPS group. Meanwhile, the expression levels of Nrf2 and Keap-1 in the LPS+Pro group showed no significant differences compared with the Control group and the LPS group, showing a trend to maintain the homeostasis of the Nrf2/Keap-1 mediated antioxidant pathway at the transcriptional level.

In the inflammatory pathway, compared with the Control group, the mRNA expression levels of *TLR4* (*P* < 0.05), *NF-κB* (*P* < 0.05), *TNF-α* (*P* < 0.05), and *IL-6* (*P* < 0.05) were significantly increased in the LPS group. Compared with the LPS group, the mRNA expression levels of *TLR4* (*P* < 0.05), *NF-κB* (*P* < 0.05), *TNF-α* (*P* < 0.05), and *IL-6* (*P* < 0.05) were significantly decreased in the LPS+Pro group.

### LPS levels and correlation analysis of the gut-liver axis

**Fig. 9:**
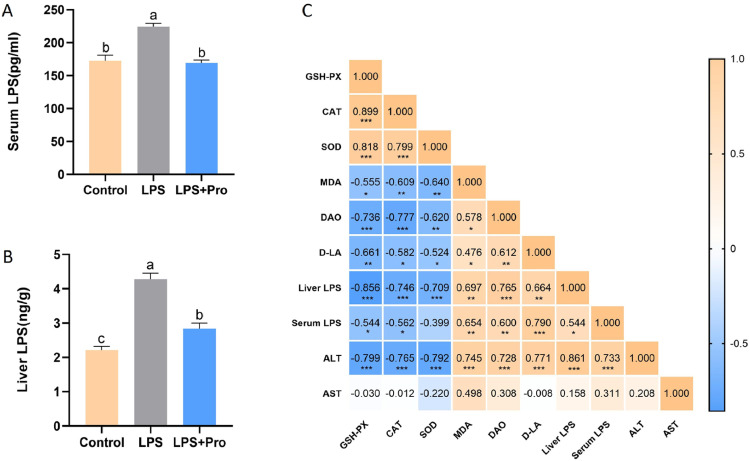
Serum and liver LPS levels in Lohmann Pink chickens on day 22 of the experiment and correlation analysis of the gut-liver axis. (A) Serum LPS concentration. (B) Liver LPS concentration. Data are presented as mean ± SEM, n = 6. Bars with different letters (a-c) indicate significant differences (*P* < 0.05). (C) Heatmap of Spearman’s rank correlations among biochemical parameters (Liver GSH-Px, Liver CAT, Liver SOD, Liver MDA, DAO, d-LA, Liver LPS, Serum LPS, ALT, AST). The color depth reflects the magnitude of the correlation coefficient, n = 6. Asterisk (*) indicates statistically significant correlations (*P<0.05; **P<0.01; ***P<0.001). Control: fed a basal diet and injected with saline on days 15, 17, and 19. LPS: fed a basal diet and injected with LPS on days 15, 17, and 19. LPS+Pro: fed a basal diet supplemented with compound probiotics and injected with LPS on days 15, 17, and 19.

Compared with the Control group, serum LPS concentration was significantly increased in the LPS group (*P* < 0.05), whereas no significant difference was observed in the LPS+Pro group (*P* > 0.05) (Fig. 9A). Hepatic LPS content was significantly elevated in both the LPS group and LPS+Pro group relative to the Control group (*P* < 0.05). However, hepatic LPS level was markedly lower in the LPS+Pro group than in the LPS group (*P* < 0.05) (Fig. 9B).

Spearman’s rank correlation analysis revealed significant associations among hepatic antioxidant status, intestinal barrier function, liver injury, and LPS levels (Fig. 9C). Liver antioxidant enzymes (GSH-Px, CAT, SOD) were strongly positively intercorrelated, and all were significantly negatively correlated with MDA, intestinal injury markers (DAO, D-LA), liver injury marker ALT, and liver LPS. Conversely, MDA was positively correlated with DAO, D-LA, ALT, and LPS levels. DAO and D-LA were positively intercorrelated, and both were strongly associated with elevated ALT and LPS concentrations. Liver and serum LPS were significantly positively correlated with each other, and both were strongly positively associated with ALT. No significant correlation was observed between AST and any other parameter.

## Discussion

Before the LPS challenge, dietary supplementation with the compound probiotics in the LPS+Pro group significantly increased ADG in the second week of feeding. Similarly, in a previous unpublished preliminary experiment in our laboratory, dietary supplementation with the same dose of the compound probiotics starting at four weeks of age in Lohmann Pink chickens (half male and half female) significantly increased ADG (Compound probiotic group: mean=14.21 g, a 40.89% increase, *P* *=* 0.029) and average daily feed intake (ADFI, Compound probiotic group: mean=55.76 g, a 22.47% increase, *P* *<* 0.01), and significantly decreased the feed-to-gain ratio (F/G, Compound probiotic group: mean=3.85, a 7.67% decrease, *P* *=* 0.024) in the third week of supplementation. One week after the first LPS challenge, ADG was markedly reduced in the LPS group, which was consistent with the performance of the LPS group during the challenge period: the LPS group showed reduced activity and feed intake, with chickens often huddling in corners and lying prostrate. In contrast, ADG in the LPS+Pro group recovered to the level of the Control group. Similarly, Chen and Yu (2021) and Jiang et al. (2024) found that dietary supplementation with 2 × 10^10^ CFU/kg, 3 × 10^10^ CFU/kg, or 6 × 10^10^ CFU/kg *Bacillus subtilis* with or without its fermented products in broilers alleviated the significant decrease in ADG caused by LPS stress. Additionally, at 6 h post the first LPS challenge, the LPS+Pro group showed a significantly lower rectal temperature than the LPS group. These results indicated that the compound probiotics exerted a protective effect against LPS-induced reduction in ADG and elevation in rectal temperature.

*Claudin-1, ZO-1*, and *Occludin* encode tight junction proteins, which are core regulators of intestinal barrier integrity and directly modulate epithelial morphology and paracellular permeability (Hu et al., 2023; Liu et al., 2023b). In the present study, composite probiotics significantly upregulated the gene expression of *Claudin-1* and *ZO-1* in the duodenum, as well as *Occludin* in the jejunum of LPS-challenged Lohmann Pink chickens. These results indicated that composite probiotics improved the molecular regulation of intestinal barrier function.

D-LA and DAO serve as specific biomarkers for intestinal mucosal integrity and barrier permeability, whose circulating concentrations are positively associated with mucosal injury (Liu et al., 2020; Sun et al., 2013). It has been reported that D-LA, endotoxin, and DAO levels were increased in intestinal mucosa damage (Sun et al., 2013). In the present study, composite probiotics significantly decreased the leakage of intestinal DAO and D-LA, which was in line with previous observations in LPS-challenged broilers supplemented with 5 × 10^9^ CFU/kg *Bacillus coagulans* (Yu et al., 2022). Meanwhile, composite probiotics significantly elevated the V/C ratio in the duodenum and jejunum, and decreased duodenal crypt depth. Histopathological examination showed that composite probiotics supplementation obviously alleviated mucosal damage in the duodenum and jejunum under LPS stress, with nearly restored villus morphology, relatively regular arrangement, and improved integrity of epithelial cells. Similarly, Jiang et al. (2024) found that dietary supplementation with 3 × 10^10^ CFU/kg *Bacillus subtilis* led to obvious recovery trends in villus length, villus width, and villus area of the jejunum and ileum in broilers compared with the LPS-challenged group. Collectively, these results demonstrated that composite probiotics attenuate LPS-induced intestinal barrier injury by reducing intestinal permeability and preserving the structural integrity of the small intestine in Lohmann Pink chickens.

Nrf2 is a key transcription factor sequestered in the cytoplasm by Keap1, which mediates its ubiquitination and degradation. It regulates cellular antioxidant responses by inducing the expression of downstream antioxidant enzymes, including GSH-Px, SOD, and CAT. These enzymes act synergistically to maintain intracellular redox homeostasis, while MDA content serves as a critical marker for assessing oxidative damage (Espinosa-Diez et al., 2015). Multiple studies have demonstrated that dietary supplementation with either *Bacillus subtilis* or *Bacillus coagulans* effectively improves systemic, intestinal, and hepatic antioxidant capacity in poultry exposed to adverse environmental stimuli, including heat stress, Salmonella infection, and LPS challenge (Jiang et al., 2024, 2025; Song et al., 2014; Zhen et al., 2018). In the present study, compound probiotics significantly mitigated the LPS-induced systemic and hepatic oxidative damage. Furthermore, compound probiotics showed a trend to maintain homeostasis of the Nrf2/Keap-1 mediated antioxidant pathway at the transcriptional level.

The TLR4/NF-κB signaling pathway acts as a central mediator in LPS-induced inflammatory responses (Luo et al., 2025). Studies using single-strain probiotics have shown that dietary supplementation with *Bacillus subtilis, Bacillus coagulans*, or *Saccharomyces cerevisiae* effectively alleviated LPS-induced systemic, intestinal, and hepatic inflammation in broilers (Chen and Yu, 2021; Jiang et al., 2025; Wang et al., 2016; Yu et al., 2022; Zhang et al., 2024). In the present study, compound probiotics significantly downregulated the hepatic TLR4/NF-κB signaling pathway at the transcriptional level, indicating that LPS-induced inflammatory responses were attenuated. These findings were consistent with the reduced hepatic inflammatory infiltration observed in histopathological sections. Collectively, compound probiotics markedly mitigated LPS-induced hepatic inflammatory infiltration and oxidative damage, preserved hepatic tissue integrity, and thus reduced the release of the liver injury marker ALT into the circulation.

To explore the effects of compound probiotics on the gut-liver axis under LPS challenge, serum and hepatic LPS levels were determined, followed by correlation analysis of biochemical indices. Correlation analysis further revealed that serum and hepatic LPS levels were significantly positively correlated with intestinal barrier damage markers and negatively correlated with hepatic antioxidant indices. On one hand, this correlation may be explained by the ability of compound probiotics to maintain intestinal integrity and reduce LPS translocation from the gut to the liver, thus alleviating LPS-induced impairment of hepatic antioxidant capacity (Liu et al., 2023a; Yu et al., 2022). On the other hand, our preliminary experiment verified that four consecutive weeks of dietary supplementation with this dose of compound probiotics significantly elevated hepatic GSH-Px, CAT, and SOD activities in 8-week-old Lohmann Pink chickens (*P* < 0.05). This suggested that compound probiotics might enhance hepatic antioxidant function, thereby attenuating oxidative damage to liver sinusoidal endothelial cells (LSECs), Kupffer cells, and hepatocytes, and ultimately maintaining hepatic LPS clearance efficiency and reducing LPS levels in the systemic circulation (Chen et al., 2024; Kumar et al., 2024; Yao et al., 2016).

As a preliminary investigation, this study integrated the protective effects of compound probiotics under LPS challenge and their potential as antibiotic alternatives. However, several limitations remain. Given the relatively small sample size and the absence of a positive control, follow-up studies with larger cohorts and a positive control are warranted to further validate and benchmark these preliminary findings. The underlying mechanisms were not fully elucidated at the protein level, which deserves in-depth investigation in future studies. The gut-liver axis interactions were inferred primarily from correlative analyses of biochemical parameters, lacking direct evidence. Future studies should further explore the associations between gut microbiota and their metabolites to more comprehensively clarify the specific mechanisms by which compound probiotics exert their protective effects.

## Conclusions

This study demonstrated that compound probiotics mitigated LPS-induced intestinal and hepatic injury, as well as growth impairment in Lohmann Pink chickens, by enhancing intestinal barrier integrity and hepatic antioxidant capacity. Mechanistically, at the transcriptional level, compound probiotics suppressed the TLR4/NF-κB inflammatory pathway and showed a trend to maintain the homeostasis of the Nrf2/Keap-1 mediated antioxidant pathway in the liver under LPS stress. These findings provided a promising strategy for antibiotic alternatives in healthy poultry breeding.

## CRediT authorship contribution statement

**Yongliang Zhang:** Writing – review & editing, Writing – original draft, Visualization, Project administration, Methodology, Data curation. **Xixi Dai:** Writing – review & editing. **Bingran Yu:** Writing – review & editing, Visualization, Methodology, Data curation. **Jia Yu:** Writing – review & editing, Methodology, Data curation. **Linlin Wang:** Writing – review & editing, Methodology. **Ruoxi Zhan:** Writing – review & editing, Visualization. **Liangqing Chen:** Writing – review & editing, Methodology. **Xing Liu:** Data curation. **Kaiqing Rao:** Writing – review & editing, Supervision, Resources, Methodology, Funding acquisition, Conceptualization.

## Disclosures

The authors declare that they have no known competing financial interests or personal relationships that could have appeared to influence the work reported in this paper.

## Appendix Supplementary materials

Image, application 1

## Footnotes

Supplementary material associated with this article can be found, in the online version, at doi:10.1016/j.psj.2026.107306.
