# Supplementary material for: Effects of compound probiotics on intestinal and liver injury in Lohmann Pink chickens challenged by lipopolysaccharide
Source: Poult Sci. 2026 Jun 17;105(10):107306. doi: 10.1016/j.psj.2026.107306 (PMC13315343; doi:10.1016/j.psj.2026.107306)
Supplement: Supplementary file 1 [file mmc1.docx]

**Table S1 Composition and nutrient levels of the basal diet (air-dry basis, %)**

| **Item** | **Content (%)** |
| --- | --- |
| **Ingredients** |  |
| Corn | 63.25 |
| Soybean meal (43% crude protein) | 30.85 |
| Wheat bran | 1.50 |
| Soybean oil | 0.50 |
| Limestone | 1.26 |
| Calcium hydrogen phosphate | 1.70 |
| L-Lysine·HCl (98%) | 0.02 |
| DL-Methionine (98%) | 0.18 |
| L-Threonine (98%) | 0.00 |
| Sodium chloride | 0.17 |
| Trace mineral premix¹ | 0.50 |
| Vitamin premix² | 0.02 |
| Choline chloride (50%) | 0.05 |
| **Total** | **100.00** |
| **Nutrient levels³** |  |
| Metabolizable energy (MJ/kg) | 11.64 |
| Crude protein | 18.50 |
| Calcium | 1.00 |
| Total phosphorus | 0.68 |
| Available phosphorus | 0.45 |
| Lysine | 1.00 |
| Methionine | 0.47 |

Footnotes

¹ The trace mineral premix provided the following per kg of diet: Fe 25 mg, Cu 5 mg, Mn 100 mg, Zn 60 mg, I 0.5 mg, Se 0.2 mg.

² The vitamin premix provided the following per kg of diet: vitamin A 8,000 IU, vitamin D₃ 2,000 IU, vitamin E 20 mg, vitamin K₃ 3.2 mg, vitamin B₁ 2 mg, vitamin B₂ 6.4 mg, vitamin B₆ 4 mg, vitamin B₁₂ 0.02 mg, niacin 40 mg, pantothenic acid 12 mg, folic acid 1 mg, biotin 0.11 mg.

³ Nutrient levels were calculated values.

The basal diet was formulated according to the *Lohmann Pink Laying Hen Management Manual* (07.22_V07-23).
